# Supplementary material for: The Business of DNA Nanotechnology: Commercialization of Origami and Other Technologies
Source: Molecules. 2020 Jan 16;25(2):377. doi: 10.3390/molecules25020377 (PMC7024392; doi:10.3390/molecules25020377)
Supplement: Supplementary file 1 [file molecules-25-00377-s001.pdf]

SUPPLEMENTARY INFORMATION

The business of DNA nanotechnology: commercialization of origami and other technologies

Katherine E. Dunn[1,*]

[1] School of Engineering, Institute for Bioengineering, University of Edinburgh, The King's Buildings, Edinburgh, EH9 3DW, Scotland, UK

* Correspondence: k.dunn@ed.ac.uk

Supplementary Data 1: Patent searches

These tables contain the results of searches on patent database Espacenet. The search string used in each case is indicated. The title and abstract of the patent applications were searched. Note that many of these patent applications will not be granted. Some will be rejected on grounds of insufficiency, or lack of novelty or patentability. The decision to include or exclude a patent application was based on an examination of the title, abstract and any diagram supplied on the Espacenet page. The full text of the patents were not examined. Patent abstracts in an unknown language were automatically translated by Espacenet into English. The translation is imperfect. Titles are given here as provided in Espacenet, without correction of obvious language issues (such as 'imagination' in place of 'imaging'). The definition of DNA nanotechnology is given in the body of the paper.

Notation used for decisions:

Excl – filtered out (off-topic)

Dup – duplicate of another entry

In – included in analysis

N/A – after cut-off date of 31/12/17

Search string = DNA nanotechnology

TITLE OF PATENT (as it appears on Espacenet)	Priority date	Decision
1. GENERATING NUCLEATION CENTERS ON NUCLEIC ACID, USEFUL FOR SUBSEQUENT METALLIZATION IN NANOTECHNOLOGY, COMPRISES INCUBATION WITH A METAL SALT THEN REDUCTION	22/06/2001	excl
2. DNA AND RNA SEQUENCING BY NANOSCALE READING THROUGH PROGRAMMABLE ELECTROPHORESIS AND NANOELECTRODE-GATED TUNNELING AND DIELECTRIC DETECTION	28/01/2002	excl
3. NUCLEOSIDE DERIVATIVE, MODIFED OLIGONUCLEOTIDE, METHOD FOR THEIR SYNTESIS AND APPLICANTIONS THEREOF	02/05/2003	excl
4. MICRO AND NANO SCALE FABRICATION AND MANUFACTURE BY SPATIALLY SELECTIVE DEPOSITION	30/06/2003	excl
5. PROBE MICROSCOPE SYSTEM SUITABLE FOR OBSERVING SAMPLE OF LONG BODY	03/09/2004	excl
6. DRUG SCREENING METHOD, USES NANOTECHNOLOGY TO APPLY MOTOR PROTEIN FILAMENTS TO LOADING ZONES ON CHIP AND IMMOBILISED MOTOR PROTEINS TO PROPEL THEM INTO NANOCHANNELS	07/09/2004	excl
7. CHEMICAL, PARTICLE, AND BIOSENSING WITH NANOTECHNOLOGY	29/09/2004	excl
8. METHOD FOR PRODUCTION OF SUPRAMOLECULAR COMPOSITES	13/04/2005	excl
9. PREPARATION AND USE FOR AFFINITY HUMAN ALBUMIN NUCLEIC ACID APTAMER	22/09/2006	excl
10. NUCLEIC ACID APTAMER WITH HIGH SPECIFICITY AND HIGH AFFINITY TO HUMAN BREAST CARCINOMA TISSUE, PREPARATION METHOD AND APPLICATION THEREOF	22/09/2006	excl
11. METHOD FOR PREPARING ANTI-CANCER MEDICAMENT CAPABLE OF CARRYING OUT AUTOMATIC SYNTHESIS AND TARGET RELEASE	08/12/2008	excl
12. METHOD OF PRODUCTION OF FULL-LENGTH HBV CORE PROTEIN CAPSIDS	14/01/2009	excl
13. PACKAGING OF MAGNETIC NANOPARTICLES INTO HBV CORE PROTEIN-FORMED CAPSIDS	15/09/2009	excl
14.METHOD OF PACKAGING BIOLOGICAL MATERIAL IN HEPATITIS B VIRAL CORE FULL LENGTH PROTEIN CAPSIDS	08/10/2009	excl
15. PEG-PLGA-PLL POLYMER AND METHOD FOR PREPARING AND USING THE SAME AS THE DRUG AND GENE CARRIER	30/12/2009	excl
16. DX TILE GROUP CAPABLE OF APPLYING MULTI-RULE TO DNA NANOTECHNOLOGY ALGORITHMIC ASSEMBLY	30/04/2010	in

17. INFLUENZA VIRUS DETECTION KIT	06/05/2010	in
18. KIT FOR DETECTING HUMAN IMMUNODEFICIENCY VIRUS	06/05/2010	dup
19. ESCHERICHIA COLI DETECTION KIT	06/05/2010	dup
20. KIT FOR DETECTING HEPATITIS B VIRUS	18/05/2010	dup
21. KIT FOR DETECTING AH1N1 INFLUENZA VIRUS	18/05/2010	dup
22. KIT FOR DETECTING HEPATITIS C VIRUS	18/05/2010	dup
23. KIT FOR DETECTING SALMONELLA SPP.	18/05/2010	dup
24. NANOTECHNOLOGY-BASED TRACE PROTEIN DETECTION METHOD	28/06/2010	in
25. METHOD FOR DETECTING POLYMORPHISM OF SINGLE NUCLEOTIDE WITH GOLD NANOPARTICLES	12/10/2010	in
26. METHOD FOR DETECTING DNA SEQUENCE THROUGH POLYMERASE CHAIN REACTION STAINING TECHNOLOGY	29/11/2010	in
27. NUCLEIC ACID SEPARATOR FOR GENE DETECTION IN TREATMENT ON PROGRESSIVE MYODYSTROPHY	06/04/2011	in
28. DETECTION METHOD OF MERCURY ELEMENT	01/12/2011	in
29. KIT FOR DETECTING H5 AVIAN INFLUENZA VIRUS	24/09/2012	dup
30. KIT FOR DETECTING H3N2 AVIAN INFLUENZA VIRUS	24/09/2012	dup
31. KIT FOR DETECTING N2 AVIAN INFLUENZA VIRUS	24/09/2012	dup
32. KIT FOR DETECTING H5N2 AVIAN INFLUENZA VIRUS	24/09/2012	dup
33. KIT FOR DETECTING AVIAN INFLUENZA VIRUS (AIV) H9N2	24/09/2012	dup
34. KIT FOR DETECTING H5N1 AVIAN INFLUENZA VIRUS	24/09/2012	dup
35. REFILLABLE DRUG DELIVERY DEVICES AND METHODS OF USE THEREOF	04/04/2014	excl
36. APPLE VIRUS DETECTION METHOD AND DETECTION KIT THEREBY	08/05/2014	in
37 METHOD OF PRODUCING NANOCOMPOSITES BASED ON GOLD NANOPARTICLES COATED WITH SHELL MADE OF SILICON OXIDE AND QUANTUM DOTS	14/08/2014	excl
38. PORTABLE DEVICE	25/03/2015	excl
39. MICROALGAE SEPARATION APPARATUS USING ULTRASONIC	31/03/2015	excl
40. METHOD FOR OBTAINING PH-SENSITIVE BRANCHED NODES OF DNA-NANOCONSTRUCTIONS	05/10/2015	in
41. PREPARATION METHOD OF DNA SUPER-LATTICE NANO MEDICINE CARRYING MOLECULE AIMING AT TUMOR THERAPY	19/11/2015	in
42. SIGNAL RESPONDING AND MONITORING METHOD IN SELF-ASSEMBLY CASCADE REACTION	17/05/2016	in
43. DESIGN METHOD OF LIGHT-ACTIVATED LOGIC CIRCUIT BASED ON DNA DOUBLE-HELIX STRUCTURE	23/05/2016	in

44. SINGLE MOLECULE DETECTION OR QUANTIFICATION USING DNA NANOTECHNOLOGY	15/06/2016	in
45. SCALABLE BIOTECHNOLOGICAL PRODUCTION OF DNA SINGLE STRAND MOLECULES OF DEFINED SEQUENCE AND LENGTH	21/09/2016	in
46. DNA MATERIAL-BASED MAGNETIC NANOMETER TREATMENT AND DIAGNOSIS REAGENT, AND PREPARATION AND APPLICATION THEREOF	07/11/2016	in
47. NANO DI-CYCLIC APTAMER PROBE AND APPLICATION THEREOF	16/12/2016	in
48. DNA STRUCTURAL ELEMENT OF ION CONCENTRATION RESPONSE AND PREPARATION AND APPLICATION THEREOF	20/12/2016	in
49. THERMOTHERAPY AND CHEMOTHERAPY INTEGRATED NANO-PARTICLES AND PREPARATION AND APPLICATION THEREOF	20/12/2016	in
50. DETECTING METHOD FOR DNA MORPHOLOGY VARIATION WITH TEMPERATURE AT INTERFACE	27/12/2016	in
51. POLYOLIGONUCLEIC ACID MOLECULE AND ITS APPLICATION IN MULTI-TARGET INTERFERENCE	22/01/2017	in
52. METHOD OF REGULATING AND CONTROLLING IN-SITE CATALYTIC POLYMERIZATION REACTION WITH BIOENZYME ON NANO MATERIAL ON THE BASIS OF DNA	22/02/2017	in
53. METHOD FOR DETERMINING SIGNAL ACQUISITION SITE UNDER SINGLE MOLECULE FLUORESCENCE OBSERVATION BASED ON DNA ORIGAMI	10/08/2017	in
54. METHOD FOR STUDYING CASCADE REACTIONS UNDER SINGLE-MOLECULE CONDITION	10/08/2017	in
55. METHOD FOR PREPARING REGULARLY-ARRANGED FLUORESCENTLY LABELED DNA-GOLD NANO-ARRAY	10/08/2017	in
56. DNA MOLECULAR TILE OR NUCLEIC ACID NANO-STRUCTURE THEREOF, AND APPLICATIONS THEREOF	21/09/2017	in
57. METHOD FOR DETECTING PROSTATE SPECIFIC ANTIGEN UNDER MONOMOLECULAR CONDITION	26/10/2017	in
58. FLUORESCENCE PRECISE QUANTIFICATION METHOD OF INTEGRITY OF DNA ORIGAMI STRUCTURE	27/10/2017	in
59. METHOD FOR CONSTRUCTING CASCADE REACTION BY USING DNA ORIGAMI STRUCTURE	07/11/2017	in
60. METHOD OF DETECTING TARGET SARCOSINE OF PROSTATIC CANCER	07/11/2017	in
61. DEVICE FOR CONTROLLING DIFFUSION OF DNA IN SOLID-STATE NANOPORE SEQUENCING EXPERIMENT BY MAGNETIC BEADS	21/11/2017	excl
62. DNA NANO-IMAGING PROBE FOR SUBCELLULAR-SCALE CELLULAR CO-LOCALIZATION AS WELL AS	28/11/2017	in

PREPARATION METHOD AND APPLICATION OF DNA NANO-IMAGING PROBE		
63. SINGLE MOLECULE DETECTION OR QUANTIFICATION BY DNA NANOTECHNOLOGY IN MICROWELLS	14/12/2017	in
64. MANUFACTURING METHOD OF DNA-BASED CONDUCTIVE COATING AND PRODUCT AND USE THEREOF	26/12/2017	in
65. PREPARATION METHOD OF DNA CONDUCTIVE FILM, AND PRODUCT AND APPLICATION THEREOF	26/12/2017	dup
66. INFORMATION ENCRYPTION METHOD BASED ON DNA NANOTECHNOLOGY	11/05/2018	n/a
67. METHOD FOR POLYMERIZATION ON DNA ORIGAMI SURFACE BASED ON DNAZYME	25/06/2018	n/a
68. METHOD FOR STUDYING INFLUENCE OF INTERFACE EFFECT ON STABILITY OF DNA TWO-DIMENSIONAL ORIGAMI STRUCTURE	27/07/2018	n/a
69. METHOD OF DETECTING MICRORNAS BASED ON ROLLING CIRCLE AMPLIFICATION AND DNA ORIGAMI	28/11/2018	n/a
70. METHOD FOR STUDYING INFLUENCE OF MAGNETIC FIELD ON STABILITY OF DNA TWO-DIMENSIONAL PAPER FOLDING STRUCTURE	13/12/2018	n/a
71. METHOD FOR CONSTRUCTING CLUSTER-SHAPED DNA NANOSTRUCTURE	28/12/2018	n/a
72. METHOD OF SIMPLY CONSTRUCTING INDETERMINATE GROWTH DNA NANO MECHANISM	28/12/2018	n/a

Search string = DNA nanostructure*

Here the asterisk is included in the search string to ensure that variants of the search term nanostructure are also included – such as ‘nanostructures’ or ‘nanostructured’.

**TITLE OF PATENT (as it appears on Espacenet)**	**Priority date**	**Decision**
1. METALLISCHE NANOSTRUKTUR AUF DER BASIS HOCHGEORDNETER PROTEINE SOWIE VERFAHREN ZU DEREN HERSTELLUNG	19/06/1996	excl
2. CATALYST WITH ENANTIOSELECTIVE ACTION HAS METALLIC NANOSTRUCTURE ON METALLIZED BIO-TEMPLATE, MODIFIED BY INCOMPATIBLE ENVIRONMENTAL CONDITIONS WITH SELF-ASSEMBLED HIGH ORDER PROTEINS OR DNA	01/02/2001	excl
3.MULTIFUNCTIONAL BIOSENSOR BASED ON ZNO NANOSTRUCTURES	06/06/2002	excl
4. HYPERBRANCHED DENDRON AND METHODS OF SYNTHESIS AND USE THEREOF	21/03/2003	excl
5. METHOD AND PRODUCTS FOR DELIVERING BIOLOGICAL MOLECULES TO CELLS USING MULTICOMPONENT NANOSTRUCTURES	24/06/2003	excl
6. NANO-PATTERNED STRUCTURE AND THEIR MANUFACTURING PROCESS	17/09/2003	excl
7. MICROCONTACT PRINTING METHOD AND APPARATUS	24/10/2003	excl
8. MOLECULAR LITHOGRAPHY WITH DNA NANOSTRUCTURES	23/03/2004	in
9. NANOPARTICLE RADIOSENSITIZERS	28/09/2004	excl
10. METHOD FOR PURIFYING AND POSITIONING CARBON NANOTUBE	22/04/2005	excl
11. SELF-ASSEMBLED NUCLEIC ACID NANOARRAYS AND USES THEREFOR	12/05/2005	in
12. APPARATUS AND METHOD FOR MANUFACTURING NANOSTRUCTURE	03/06/2005	excl
13. SURFACE-ENHANCED SPECTROSCOPIC METHOD, FLEXIBLE STRUCTURED SUBSTRATE, AND METHOD OF MAKING THE SAME	14/07/2005	excl
14. MICROCAPSULE NANOTUBE DEVICES FOR TARGETED DELIVERY OF THERAPEUTIC MOLECULES	22/11/2005	excl
15. METHOD FOR SELF-ASSEMBLY OF ARBITRARY METAL PATTERNS ON DNA SCAFFOLDS	26/10/2006	in
16. METHOD AND SYSTEM FOR ASSEMBLY OF MACROMOLECULES AND NANOSTRUCTURES	15/03/2007	in
17. METHOD AND SYSTEM FOR ASSEMBLY OF MACROMOLECULES AND NANOSTRUCTURES	15/03/2007	dup
18. METHOD FOR CONSTRUCTING COMPLICATED NANO FORM BY SUING DNA MOLECULE	22/03/2007	in
19. SEQUENCING SINGLE MOLECULES USING SURFACE-ENHANCED RAMAN SCATTERING	30/03/2007	excl

20. METHOD FOR PRODUCING METAL OXIDE FIBERS	27/04/2007	excl
21. APPARATUS, SYSTEM, AND METHOD FOR DNA SHADOW NANOLITHOGRAPHY	01/08/2007	in
DNA-PYRENE COMPLEXES, FLUORESCENT DNA NANOSTRUCTURES AND MANUFACTURING METHOD OF THE SAME	03/08/2007	in
22. BIOSENSOR USING NANOSCALE MATERIAL AS TRANSISTOR CHANNEL, AND METHOD OF FABRICATING THE SAME	17/09/2007	excl
23. DNA MICROARRAY HAVING HAIRPIN PROBES TETHERED TO NANOSTRUCTURED METAL SURFACE	05/11/2007	in
24. METHODS AND KITS FOR NUCLEIC ACID SEQUENCING	03/09/2008	excl
25. DESIGN, SYNTHESIS AND USE OF SYNTHETIC NUCLEOTIDES COMPRISING CHARGE MASS TAGS	03/09/2008	excl
26. LARGE-SCALE PREPARATION METHOD OF HYPERFINE NANOSTRUCTURE AND USE THEREOF	10/09/2008	excl
27. NOVEL DNA NANOSTRUCTURES THAT PROMOTE CELL-CELL INTERACTION AND USE THEREOF	03/10/2008	in
28. METHOD FOR MEASURING BIOMOLECULES USING LOCALIZED SURFACE PLASMON RESONANCE	25/02/2009	excl
29. BIOMOLECULAR SENSING WITH METAL-NANOSTRUCTURES	02/04/2009	excl
30. BIO SENSING CHIP AND METHOD FOR MANUFACTURING OF IT	29/05/2009	excl
31. METHOD OF PREPARING POLYPEPTIDES FOR CRYO-TRANSMISSION ELECTRON MICROSCOPY (CRYO-EM) AND STRUCTURE DETERMINATION USING DNA LATTICES	31/07/2009	in
32. NANOSTRUCTURED DEVICES INCLUDING ANALYTE DETECTORS, AND RELATED METHODS	04/11/2009	excl
33. ELECTROCHEMICAL DETECTION METHOD FOR DNA THREE-DIMENSIONAL NANOSTRUCTURE PROBE	09/03/2010	in
34. MATERIALS AND METHODS FOR STABILIZING NANOPARTICLES IN SALT SOLUTIONS	22/03/2010	excl
35. SHORT-ROD-SHAPED LEAD SULFIDE NANOSTRUCTURED MATERIAL AND PREPARATION METHOD THEREOF	09/09/2010	excl
36. MULTI-SPOT COPPER-CAPPED NANOSTRUCTURE ARRAY BIOCHIP USING COPPER BINDING PEPTIDE AND PREPARING METHOD THEREOF	30/09/2010	excl
37. ENZYME-MEDIATED ASSIMILATION OF DNA-FUNCTIONALIZED SINGLE-WALLED CARBON NANOTUBES (SWNTS)	06/10/2010	excl
38. METHOD AND APPARATUS FOR CONTROLLING PROPERTIES OF NUCLEIC ACID NANOSTRUCTURES	22/12/2010	in

39. A THREE-DIMENSIONAL NANOSTRUCTURED ARRAY OF PROTEIN NANOPARTICLES	07/03/2011	in
40. DNA SEQUENCING EMPLOYING NANOMATERIALS	14/04/2011	excl
REMOTE TRANSMISSION OF ELECTROMAGNETIC SIGNALS INDUCING NANOSTRUCTURES AMPLIFIABLE INTO A SPECIFIC DNA SEQUENCE	15/04/2011	excl
41. FINITE FULLY ADDRESSABLE NUCLEIC ACID NANOSTRUCTURES AS NANOCARRIERS FOR DELIVERY OF PHARMACEUTICALS	04/05/2011	in
42. CARBON FIELD EFFECT TRANSISTORS HAVING CHARGED MONOLAYERS TO REDUCE PARASITIC RESISTANCE	10/05/2011	excl
43. LAYER-BY-LAYER SURFACE FUNCTIONALIZATION OF CATALYST-FREE FULLERENE NANOSTRUCTURES AND THE APPLICATIONS THEREOF	12/09/2011	excl
44. CARBON NANOSTRUCTURE ELECTROCHEMICAL SENSOR AND METHOD	12/09/2011	excl
45. LAYER-BY-LAYER SURFACE FUNCTIONALIZATION OF CATALYST-FREE FULLERENE NANOSTRUCTURES AND THE APPLICATIONS THEREOF	18/10/2011	excl
46. DEVELOPMENT OF NUCLEIC ACID GEL MATRIX FOR CELL-FREE PROTEIN SYNTHESIS OF CELL NUCLEUS REPLICATE, AND METHOD FOR PRODUCING SAME	17/11/2011	in
47. DNA-LINKED NANOPARTICLE BUILDING BLOCKS FOR NANOSTRUCTURE ASSEMBLY AND METHODS OF PRODUCING THE SAME	30/11/2011	in
48. METHOD FOR DNA DEFINED ETCHING OF A GRAPHENE NANOSTRUCTURE	19/01/2012	in
49. NOVEL DNA-ORIGAMI NANOVACCINES	06/02/2012	in
50. SELECTIVE NUCLEIC ACID AMPLIFICATION FROM NUCLEIC ACID POOLS	09/02/2012	in
51. USES OF IDED NANOSTRUCTURES IN NUCLEIC ACID TECHNOLOGY	19/02/2012	excl
52. GENE DELIVERY SYSTEM COMPRISING BRANCHED POLYETHYLENEIMINE AND USES THEREOF	27/02/2012	excl
53. NEW LITHOGRAPHIC METHOD	05/03/2012	excl
54. NANOSTRUCTURE COATED WITH A TWIST-STRAINED DOUBLE-STRANDED CIRCULAR DEOXYRIBONUCLEIC|ACID (DNA), METHOD FOR MAKING AND USE	30/04/2012	excl
55. NANOPARTICLES IN THE SHAPE OF NANOSNOWMAN WITH A HEAD PART AND A BODY PART, A PREPARATION METHOD THEREOF AND A DETECTION METHOD USING THE SAME	04/05/2012	in

56. CONTRAST MEDIUM COMPOSITION AND METHOD OF BIO IMAGINATION USING THE SAME	07/03/2013	in
57. NOVEL NICOTINE DNA VACCINES	15/03/2013	in
58. RAPID AND HIGHLY FIELDABLE VIRAL DIAGNOSTIC	15/03/2013	excl
59. METHODS FOR OBTAINING INFORMATION FROM SINGLE CELLS WITHIN POPULATIONS USING DNA ORIGAMI NANOSTRUCTURES WITHOUT THE NEED FOR SINGLE CELL SORTING	12/06/2013	in
60. COMPOSITIONS AND METHODS RELATING TO NUCLEIC ACID-PROTEIN COMPLEXES	10/07/2013	in
61. FABRICATION OF HIERARCHICAL SILICA NANOMEMBRANES AND USES THEREOF FOR SOLID PHASE EXTRACTION OF NUCLEIC ACIDS	05/08/2013	excl
62. METHOD FOR PREPARING METAL NANOSTRUCTURE BASED ON BIOMOLECULES	13/08/2013	in
63. ENGINEERING DNA ASSEMBLY IN VIVO AND METHODS OF MAKING AND USING THE REVERSE TRANSCRIPTASE TECHNOLOGY	26/09/2013	in
64. OPTICAL LOGIC GATES AND METHOD FOR GENERATING LOGIC SIGNALS USING DNA BASED NANOSTRUCTURE	11/11/2013	in
65. METHOD FOR USING POLYPEPTIDE-MEDIATED DNA NANOSTRUCTURE AS ANTITUMOR DRUG CARRIER	27/11/2013	in
66. NATURAL RUBBER LATEX PRESERVATIVE WITH ZINC OXIDE NANOSTRUCTURES	20/12/2013	excl
67. NANOSTRUCTURES COVERED WITH SPECIES HAVING PHOTOTHERMALLY CLEAVABLE LINKERS FOR CONTROLLED DELIVERY OF CARGO	26/12/2013	excl
68. NONDESTRUCTIVE COLLECTION OF EVIDENCE	10/02/2014	excl
69. METHOD FOR MODELING 5' COHESIVE END NUCLEIC ACID SEQUENCE OF BRANCHED DNA NANOSTRUCTURE WITH ENHANCED YIELD AND MINIMIZING ERROR	01/04/2014	in
70. SELF ASSEMBLING NUCLEIC ACID NANOSTRUCTURES	03/04/2014	in
71. SELF-ASSEMBLY OF DNA ORIGAMI: A NEW DIAGNOSTIC TOOL	29/04/2014	in
72. SELF-ASSEMBLED PEPTIDE NANOSTRUCTURES BY EXPLOITING CONFORMATIONAL CHANGE, BIOSENSOR USING THE SAME AND DETECTION METHOD OF BIOMOLECULES USING THE SAME	14/05/2014	excl
73. SCALABLE NUCLEIC ACID-BASED NANOFABRICATION	22/05/2014	in
74. DETECTION METHOD USING DNA NANO-ORIGAMI STRUCTURE AS SIGNAL AMPLIFICATION PROBE	28/05/2014	in

75. METHOD FOR REDUCING BACKGROUND SIGNAL OF ELECTROCHEMICAL SENSOR AND SENSOR UTILIZING METHOD	30/05/2014	excl
76. MECHANOCHEMICAL PLATFORM AND SENSING METHODS USING DNA ORIGAMI NANOSTRUCTURES	06/06/2014	in
PREPARATION AND APPLICATION OF BIOLOGICAL NANOMETER CONTAINER TO TREATMENT OF CANCER	11/07/2014	in
77. IMPROVED NANOPORE PLASMONIC ANALYSER	15/09/2014	excl
78. ARTIFICIAL BACTERIOPHAGE BASED ON CARBON NANOSTRUCTURES FOR SUPPLYING MEDICAMENTS.	10/10/2014	excl
79. ELECTROCHEMICAL SENSOR BASED ON STEM-AND-LOOP STRUCTURED PROBE AND PREPARATION METHOD OF ELECTROCHEMICAL SENSOR	15/10/2014	in
80. METHOD FOR MASS PRODUCTION OF NUCLEIC ACID NANOSTRUCTURE AND USE THEREOF IN DRUG DELIVERY SYSTEMS	21/10/2014	in
81. METHOD FOR CONTROLLING SELF ASSEMBLY OF GOLD NANOPARTICLES BY VIRTUE OF DNA (DEOXYRIBONUCLEIC ACID) TETRAHEDRON AND I-MOTIF STRUCTURE	19/11/2014	in
82. NOVEL TETRAHEDRON NANOSTRUCTURE CONSISTING OF DNA-RNA HYBRID OR RNA	02/03/2015	in
83. NOVEL DNA-RNA HYBRID REGULAR TETRAHEDRON STRUCTURE OR RNA TETRAHEDRON STRUCTURE	02/03/2015	dup
84. SINGLE-STRANDED DNA NANOSTRUCTURES	07/03/2015	in
85. DNA METHOD FOR PREPARING DNA TEMPLATE FOR NANO-STRUCTURE FABRICATION	11/06/2015	in
86. NANOSTRUCTURE FOR DETECTING CELL-FREE DNA USING CONDUCTIVE POLYMER AND THE USE THEREOF	23/06/2015	excl
87. METHOD FOR POINT MUTATION DIAGNOSING IN NATIVE DNA USING GRAPHENE OXIDE	25/11/2015	excl
88. METHOD FOR PHASE TRANSITION OF HYDROPHOBIC NANOPARTICLES BY USING DNA NANOSTRUCTURE	26/11/2015	in
89. NANOSTRUCTURES WITH CATALYTIC ACTIVITY	27/11/2015	in
90. RAPID GENE SENSORS FROM CARBON NANOTUBE-DNA SYSTEMS	15/01/2016	excl
91. BIOMOLECULE DETECTING SENSOR METHOD FOR MANUFACTURING THE SAME AND BIOMOLECULE DETECTING METHOD	10/03/2016	in
92. DNA SELF-ASSEMBLED NANOSTRUCTURES CONTAING LIPID-DNA AND IMMUNE MODULATORY MOLECULES AND PHARMACEUTICAL COMPOSITION FOR THE	10/03/2016	in

TREATMENT OR PREVENTION OF CANCER INFECTIOUS DISEASES OR IMMUNE DISEASES COMPRISING THE SAME AS ACTIVE INGREDIENT		
92. DNA SEQUENCING BY SYNTHESIS WITH NUCLEOTIDE ANALOGUES AND RAMAN DETECTION	04/04/2016	excl
NOVEL NUCLEIC ACID NETWORK NANOSTRUCTURE BASED ON TOPOISOMERASE AND PREPARATION METHOD THEREOF	19/04/2016	in
93. STABLE NANOSCALE NUCLEIC ACID ASSEMBLIES AND METHODS THEREOF	27/04/2016	in
94. SINGLE MOLECULE DETECTION OR QUANTIFICATION USING DNA NANOTECHNOLOGY	15/06/2016	in
95. STRUCTURE ASSISTED DIRECTED EVOLUTION OF MULTIVALENT APTAMERS	09/08/2016	in
96. METHOD FOR PREPARING FINITE OR INFINITE DNA NANOSTRUCTURES THROUGH SELF-ASSEMBLING OF NUCLEIC ACID STRUCTURAL UNITS	31/08/2016	in
97. TDNS-AS1411-NUCLEIC ACID DRUG NANOCOMPOSITE BASED DRUG DELIVERY SYSTEM AND PREPARATION METHOD THEREOF	02/11/2016	in
98. HYBRID NANOPORES WITH ANNULAR DNA NANOSTRUCTURES	30/11/2016	in
99. HYPERBRANCHED NANOSTRUCTURE-BASED SURFACE-ENHANCED RAMAN BIOLOGICAL MOLECULE DETECTION METHOD	01/12/2016	excl
100. CELL-FREE DNA MAGNETIC NANOSTRUCTURE FOR DETECTING AND ISOLATING CIRCULATING CELL-FREE DNA COMPRISING CONDUCTIVE POLYMERS CONTAINING MAGNETIC NANOPARTICLES AND CATIONIC POLYMERS	23/12/2016	excl
101. SYSTEMS AND METHODS FOR DETERMINING MOLECULAR MOTION	05/01/2017	in
102. NANOSTRUCTURES TO CONTROL DNA STRAND ORIENTATION AND POSITION LOCATION FOR TRANSVERSE DNA SEQUENCING	01/02/2017	excl
103. OPTICAL AND OPTO-ACOUSTIC INTEGRATION DUAL-MODE MOLECULAR IMAGE PROBE AS WELL AS PREPARATION METHOD AND APPLICATION THEREOF	16/02/2017	in
104. STIMULI-RESPONSIVE TYPE NUCLEIC ACID NANOSTRUCTURE CARRIER CHIRAL NOBLE METAL NANOCOMPOSITE AND PREPARATION METHOD THEREOF AND APPLICATION	20/02/2017	in
105. POLYMER-IRON OXIDE NANO-COMPLEX, USES THEREOF AND PREPARATION METHOD THEREOF	21/02/2017	excl
106. DNA ORIGAMI NANOSTRUCTURES FOR TREATMENT OF ACUTE KIDNEY INJURY	09/03/2017	in

107. DNA DNA NANOSTRUCTURE FOR CONTROLLING FREEZING AND MANUFACTURING METHOD OF THE SAME	27/03/2017	in
108. DNA NANOSTRUCTURE PATTERNED TEMPLATES	31/05/2017	in
109. METHOD FOR STEPWISE ASSEMBLING DNA ORIGAMI UNITS	02/06/2017	in
COMPOSITIONS AND METHODS FOR DETECTING VIRAL NUCLEIC ACIDS	23/06/2017	in
110. BRAIN TARGETING AGENT AS WELL AS PREPARATION METHOD AND APPLICATION THEREOF	18/07/2017	in
111. HIGHLY SENSITIVE AND DECOMPOSABLE QUANTUM DOT NANOSPHERE PROBE AND PREPARATION METHOD THEREOF	26/07/2017	in
112. FORMULATION OF 18-BETA-GLYCYRRHETINIC ACID IN COMBINATION WITH RESVERATROL AND METFORMIN, USES, AND METHOD FOR MANUFACTURING SAME	22/08/2017	excl
113. MATERIALS AND METHODS FOR EFFECTIVE IN VIVO DELIVERY OF DNA NANOSTRUCTURES TO ATHEROSCLEROTIC PLAQUES	28/08/2017	in
114. DNA - DNA-CONTAINING POLYMER-IRON OXIDE NANOCOMPLEX AND METHOD FOR REGULATING GENE EXPRESSION USING THE SAME	11/10/2017	in
115. NUCLEIC ACID AND OTHER COMPOSITIONS AND METHODS FOR THE MODULATION OF CELL MEMBRANES	02/11/2017	in
116. MOLECULAR DETECTION VIA PROGRAMMABLE SELF-ASSEMBLY	10/11/2017	excl
117. DNA NANOROBOT AND METHODS OF USE THEREOF	07/12/2017	in
118. DNA NANOROBOT AND METHODS OF USE THEREOF	07/12/2017	dup
119. COMPOSITIONS FOR THE TREATMENT OR PREVENTION OF NEURODEGENERATIVE DISORDERS, IN PARTICULAR PARKINSON`S DISEASE	11/12/2017	excl
120. DNA RNA DNA RNA DETECTING APPARATUS FOR DNA OR RNA KIT COMPRISING SAME AND SENSING METHOD FOR DNA OR RNA	12/12/2017	excl
121. DETECTING APPARATUS FOR DNA OR RNA, KIT COMPRISING SAME, AND SENSING METHOD FOR DNA OR RNA	12/12/2017	dup
122. SINGLE MOLECULE DETECTION OR QUANTIFICATION BY DNA NANOTECHNOLOGY IN MICROWELLS	14/12/2017	in
123. SIGNAL AMPLIFICATION USING NUCLEIC ACID NANOSTRUCTURE LINKED BY GOLD NANOPARTICLE AND ITS APPLICATIONS	29/12/2017	in

124. GUIDED MAGNETIC NANOSTRUCTURES FOR TARGETED AND HIGH-THROUGHPUT INTRACELLULAR DELIVERY	29/01/2018	n/a
125. DNA-CHIMERIC ANTIGEN RECEPTOR T CELLS FOR IMMUNOTHERAPY	02/02/2018	n/a
126. MULTIMODAL NEEDLE	07/03/2018	n/a
127. METAL PATTERNS BASED ON DNA NANOSTRUCTURE AS WELL AS PREPARATION METHOD AND APPLICATION OF METAL PATTERNS	14/03/2018	n/a
128. METHOD FOR SIMULTANEOUSLY DETECTING VARIOUS MICRO RNA (RIBONUCLEIC ACIDS) THROUGH COMBINATION OF ISOTHERMAL AMPLIFICATION AND FRET (FLUORESCENCE RESONANCE ENERGY TRANSFER) OF QD (QUANTUM DOT)	04/04/2018	n/a
129. MULTI-FUNCTIONAL CANCER DRUG DELIVERY NANODEVICE FOR PRECISION MEDICINE	10/04/2018	n/a
130. ASPNA (ANTISENSE PEPTIDE NUCLEIC ACID)-TDN (TETRAHEDRAL DNA NANOSTRUCTURE) CARRIER COMPOUND AS WELL AS PREPARATION METHOD AND APPLICATION THEREOF	13/04/2018	n/a
131. METHOD FOR POLYMERIZATION ON DNA ORIGAMI SURFACE BASED ON DNAZYME	25/06/2018	n/a
132. DNA ORIGAMI NANOSTRUCTURE-BASED METHOD FOR MANUFACTURING MASK	29/06/2018	n/a
133. METHOD FOR ENABLING MULTI-STAGE TARGETING MOLECULE-MODIFIED GOLD NANOCAGE COMPLEX TO ENTER TARGET TUMOR CELL	25/10/2018	n/a
134. ELECTROCHEMICAL SENSOR FOR DETECTING MICRORNA-21, PREPARATION METHOD AND APPLICATION THEREOF	13/12/2018	n/a
135. METHOD FOR CONSTRUCTING CLUSTER-SHAPED DNA NANOSTRUCTURE	28/12/2018	n/a
136. TELOMERASE ACTIVITY DETECTING METHOD BASED ON FLUORESCENCE RESONANCE ENERGY TRANSFER (FRET)	04/01/2019	n/a
137. PH-INDUCED DRUG SUSTAINED-RELEASE DEOXYRIBONUCLEIC ACID (DNA) NANOSTRUCTURE AS WELL AS PREPARATION METHOD AND APPLICATION THEREOF	11/02/2019	n/a
138. METHOD FOR SIMULTANEOUS DETECTION OF TWO MIRNAS WITH WAVELENGTH TRANSFER MOLECULAR BEACON	07/03/2019	n/a
139. DNA TETRAHEDRAL STRUCTURE, PREPARATION METHOD AND APPLICATION THEREOF	26/04/2019	n/a

Search string: DNA origami

TITLE OF PATENT (as it appears on Espacenet)	Priority date	Decision
1. NUCLEIC ACID NANOTUBE LIQUID CRYSTALS AND USE FOR NMR STRUCTURE DETERMINATION OF MEMBRANE PROTEINS	21/04/2006	in
2. CONTROLLABLE DISTRIBUTION METHOD OF GOLD NANOPARTICLES ON DNA ORIGAMI CHIP	12/10/2010	in
3. CONTROLLABLE DISTRIBUTION METHOD OF GOLD NANOPARTICLES ON DNA ORIGAMI CHIP	12/10/2010	dup
4. METHOD FOR DETECTING POLYMORPHISM OF SINGLE NUCLEOTIDE WITH GOLD NANOPARTICLES	12/10/2010	dup
5. CONTROLLABLE DISTRIBUTION METHOD OF GOLD NANOPARTICLES ON DNA ORIGAMI CHIP	12/10/2010	dup
6. DNA ORIGAMI DEVICES	04/11/2010	in
7. METHOD AND APPARATUS FOR CONTROLLING PROPERTIES OF NUCLEIC ACID NANOSTRUCTURES	22/12/2010	in
8. REAL TIME IN SITU CHARACTERIZATION METHOD FOR SINGLE BIOMOLECULAR REACTION	22/03/2011	in
9. METHOD FOR ADSORBTION OF VARIOUS BIOMATERIALS TO CHEMICALLY MODIFIED GRAPHENE	19/10/2011	excl
10. NOVEL DNA-ORIGAMI NANOVACCINES	06/02/2012	in
11. SPATIAL SEQUENCING OF NUCLEIC ACIDS USING DNA ORIGAMI PROBES	05/06/2012	in
12. DNA-ORIGAMI-BASED STANDARD	22/08/2012	in
13. CALIBRATION SAMPLE FOR CALIBRATION OF THREE-DIMENSIONAL RESOLUTION OF MEASUREMENT DEVICE, E.G. FLUORESCENCE MICROSCOPE FOR SUPER-RESOLUTION FLUORESCENCE MICROSCOPY, COMPRISES STRUCTURES BASED ON DNA ORIGAMI, WHICH HAVE TWO MARKER MOLECULES	22/08/2012	dup
14. BIOMOLECULE AFFINITY CONSTANT DETERMINATION METHOD BASED ON DNA (DEOXYRIBONUCLEIC ACID) ORIGAMI	15/10/2012	in
15. A METHOD TO PCR AMPLIFY MULTIPLE SEQUENCE ELEMENTS THAT ARE DISTANTLY LOCATED ON A SINGLE NUCLEIC ACID TOGETHER INTO A SINGLE AMPLICON	13/12/2012	in
16. METHODS FOR OBTAINING INFORMATION FROM SINGLE CELLS WITHIN POPULATIONS USING DNA ORIGAMI NANOSTRUCTURES WITHOUT THE NEED FOR SINGLE CELL SORTING	12/06/2013	in
17. COMPOSITIONS AND METHODS RELATING TO NUCLEIC ACID-PROTEIN COMPLEXES	10/07/2013	in
18. NUCLEIC ACID NANO STRUCTURE FOR CARRYING ANTITUMOR DRUGS, PREPARATION METHOD AND APPLICATIONS THEREOF	13/08/2013	in

19. SELF-ASSEMBLY OF DNA ORIGAMI: A NEW DIAGNOSTIC TOOL	29/04/2014	in
20. DETECTION METHOD USING DNA NANO-ORIGAMI STRUCTURE AS SIGNAL AMPLIFICATION PROBE	28/05/2014	in
21. MECHANOCHEMICAL PLATFORM AND SENSING METHODS USING DNA ORIGAMI NANOSTRUCTURES	06/06/2014	in
22. SITE-SPECIFIC IMMOBILIZATION OF DNA ORIGAMI STRUCTURES ON SOLID SUBSTRATES	14/10/2014	in
23. PAIN-RELIEF ACTIVE POLYPEPTIDE SCREENING METHOD	24/06/2015	excl
24. METHOD FOR SIMULATION OF DNA NANO ORIGAMI STRUCTURE AS DRUG CARRIER BY DAPI EMBEDDING AND RELEASE	26/06/2015	in
25. A PREPARATION METHOD FOR A DNA ORIGAMI BASED CARRIER SYSTEM	22/01/2016	in
26. METHOD FOR DETECTING INFLUENCE OF MECHANICAL FORCE ON INTERACTION OF DNA (DEOXYRIBOSE NUCLEIC ACID) AND DNA POLYMERASE	05/02/2016	in
27. SAMPLE PREPARATION METHOD EMPLOYING AFM (ATOMIC FORCE MICROSCOPE) FOR SINGLE ANTIBODY MOLECULE IMAGING	07/04/2016	in
28. METHOD FOR CONSTRUCTING DOLMEN STRUCTURE BASED ON DNA ORIGAMI TEMPLATE AND GOLD NANORODS	22/12/2016	in
29. SYSTEMS AND METHODS FOR DETERMINING MOLECULAR MOTION	05/01/2017	in
30. OPTICAL AND OPTO-ACOUSTIC INTEGRATION DUAL-MODE MOLECULAR IMAGE PROBE AS WELL AS PREPARATION METHOD AND APPLICATION THEREOF	16/02/2017	in
31. METHOD OF REGULATING AND CONTROLLING IN-SITE CATALYTIC POLYMERIZATION REACTION WITH BIOENZYME ON NANO MATERIAL ON THE BASIS OF DNA	22/02/2017	excl
32. DNA ORIGAMI NANOSTRUCTURES FOR TREATMENT OF ACUTE KIDNEY INJURY	09/03/2017	in
33. DNA MOLECULAR LOGIC GATE BASED ON DNA ORIGAMI AND CONSTRUCTION METHOD THEREOF	23/03/2017	in
34. NOVEL MICRO-ENVIRONMENT BIOLOGICAL MACROMOLECULE GENERAL OSCILLATOR AS WELL AS SYNTHESIS METHOD AND APPLICATION THEREOF	24/04/2017	in
35. METHOD FOR STEPWISE ASSEMBLING DNA ORIGAMI UNITS	02/06/2017	in
36. DNA ORIGAMI-BASED CONSTRUCTION METHOD AND APPLICATION OF PRECISE RECOGNITION TARGETED NANO-CARRIER	27/06/2017	in

37. PROBE MACHINE ACHIEVEMENT METHOD AND DEVICE BASED ON DNA COMPUTING	17/07/2017	in
38. NANOPROBE USED FOR SPECIFIC RECOGNITION OF DNA SEQUENCES, AND APPLICATION METHOD THEREOF	19/07/2017	in
39. METHOD FOR DETERMINING SIGNAL ACQUISITION SITE UNDER SINGLE MOLECULE FLUORESCENCE OBSERVATION BASED ON DNA ORIGAMI	10/08/2017	in
40. METHOD FOR STUDYING CASCADE REACTIONS UNDER SINGLE-MOLECULE CONDITION	10/08/2017	in
41. FLUORESCENCE PRECISE QUANTIFICATION METHOD OF INTEGRITY OF DNA ORIGAMI STRUCTURE	27/10/2017	in
42. METHOD FOR CONSTRUCTING CASCADE REACTION BY USING DNA ORIGAMI STRUCTURE	07/11/2017	dup
43. METHOD OF DETECTING TARGET SARCOSINE OF PROSTATIC CANCER	07/11/2017	in
44. MICROBIAL PRODUCTION OF PURE SINGLE STRANDED NUCLEIC ACIDS	10/11/2017	in
45. DNA NANO-IMAGING PROBE FOR SUBCELLULAR-SCALE CELLULAR CO-LOCALIZATION AS WELL AS PREPARATION METHOD AND APPLICATION OF DNA NANO-IMAGING PROBE	28/11/2017	in
46. DNA NANOROBOT AND METHODS OF USE THEREOF	07/12/2017	in
47. DNA NANOROBOT AND METHODS OF USE THEREOF	07/12/2017	dup
48. DNA ORIGAMI NANOPARTICLE DELIVERY OF PROGRAMMED CHROMOSOME BREAKAGE MACHINERY	22/12/2017	in
49. MANUFACTURING METHOD OF DNA-BASED CONDUCTIVE COATING AND PRODUCT AND USE THEREOF	26/12/2017	in
50. PREPARATION METHOD OF DNA CONDUCTIVE FILM, AND PRODUCT AND APPLICATION THEREOF	26/12/2017	dup
51. MOLECULAR LOGIC GATE BASED ON DNA ORIGAMI AND NANO METAL PARTICLE FLUORESCENT PROBE	27/12/2017	in
52. APPLICATION OF DNA NANORIBBON IN APOPTOSIS RESISTANCE AND PREPARATION METHOD OF DNA NANORIBBON	29/01/2018	n/a
53. DNA ORIGAMI BEADS FOR FLUORESCENCE QUANTIFICATION IN MICROFLUIDICS	02/02/2018	n/a
54. MULTI-FUNCTIONAL CANCER DRUG DELIVERY NANODEVICE FOR PRECISION MEDICINE	10/04/2018	n/a
55. INFORMATION ENCRYPTION METHOD BASED ON DNA NANOTECHNOLOGY	11/05/2018	n/a

56. PREPARATION METHOD OF SEQUENCE AND LENGTH CUSTOMIZED CIRCULAR SINGLE-STRANDED DNA (DEOXYRIBONUCLEIC ACID) AND APPLICATION OF SEQUENCE AND LENGTH CUSTOMIZED CIRCULAR SINGLE-STRANDED DNA TO DNA ORIGAMI	07/06/2018	n/a
57. METHOD FOR POLYMERIZATION ON DNA ORIGAMI SURFACE BASED ON DNAZYME	25/06/2018	n/a
58. DNA ORIGAMI NANOSTRUCTURE-BASED METHOD FOR MANUFACTURING MASK	29/06/2018	n/a
59. NANO-GOLD CUBE SPECIFIC MODIFICATION METHOD	18/07/2018	n/a
60. METHOD FOR STUDYING INFLUENCE OF INTERFACE EFFECT ON STABILITY OF DNA TWO-DIMENSIONAL ORIGAMI STRUCTURE	27/07/2018	n/a
61. SECURE COMMUNICATION METHOD FOR PROVIDING MULTIPLE INFORMATION PROTECTION BASED ON DNA ORIGAMI	02/08/2018	n/a
62. ACCURATE SINGLE-MOLECULE FORCE SPECTROSCOPY METHOD WITH HIGH THROUGHPUT	22/11/2018	n/a
63. METHOD OF DETECTING MICRORNAS BASED ON ROLLING CIRCLE AMPLIFICATION AND DNA ORIGAMI	28/11/2018	n/a
64. METHOD FOR FORMING DIMER STRUCTURE BY ASSEMBLING DNA ORIGAMI TEMPLATE AND NANOMETER GOLD CUBE BASED ON SURFACE ENHANCED RAMAN EFFECT	25/04/2019	n/a

Search string: nucleic acid nanostructure*

Here the asterisk is included in the search string to ensure that variants of the search term nanostructure are also included – such as ‘nanostructures’ or ‘nanostructured’.

TITLE OF PATENT (as it appears on Espacenet)	Priority date	Decision
1. METHODS FOR THE ELECTRONIC ASSEMBLY AND FABRICATION OF DEVICES	07/11/1991	in
2. NANOPARTICLES HAVING OLIGONUCLEOTIDES ATTACHED THERETO AND USES THEREFOR	29/07/1996	in
3. NANOPARTICLES HAVING OLIGONUCLEOTIDES ATTACHED THERETO AND USES THEREFOR	29/07/1996	dup
4. NANOPARTICLES HAVING OLIGONUCLEOTIDES ATTACHED THERETO AND USES THEREFOR	29/07/1996	dup
5. MOVEMENT OF BIOMOLECULE-COATED NANOPARTICLES IN AN ELECTRIC FIELD	29/07/1996	in
6. NANOPARTICLES HAVING OLIGONUCLEOTIDES ATTACHED THERETO AND USES THEREFOR	29/07/1996	dup
7. NANOPARTICLES HAVING OLIGONUCLEOTIDES ATTACHED THERETO AND USES THEREFOR	29/07/1996	dup
8. NANOPARTICLES HAVING OLIGONUCLEOTIDES ATTACHED THERETO AND USES THEREFOR	29/07/1996	dup
9. NANOPARTICLES HAVING OLIGONUCLEOTIDES ATTACHED THERETO AND USES THEREFOR	29/07/1996	dup
10. NANOPARTICLES HAVING OLIGONUCLEOTIDES ATTACHED THERETO AND USES THEREFOR	29/07/1996	dup
11. NANOPARTICLES HAVING OLIGONUCLEOTIDES ATTACHED THERETO AND USES THEREFOR	29/07/1996	dup
12. NANOPARTICLES HAVING OLIGONUCLEOTIDES ATTACHED THERETO AND USES THEREFOR	25/06/1999	dup
13. NANOPARTICLES HAVING OLIGONUCLEOTIDES ATTACHED THERETO AND USES THEREFOR	28/03/2000	dup
14. NANOPARTICLES HAVING OLIGONUCLEOTIDES ATTACHED THERETO AND USES THEREFOR	11/08/2000	dup
15. NANOPARTICLES HAVING OLIGONUCLEOTIDES ATTACHED THERETO AND USES THEREFOR	08/12/2000	dup
16. NANOPARTICLES HAVING OLIGONUCLEOTIDES ATTACHED THERETO AND USES THEREFOR	09/10/2001	dup
17. NANOSTRUCTURES CONTAINING PNA JOINING OR FUNCTIONAL ELEMENTS	21/02/2002	excl
18. HYBRIDIZATION SIGNAL AMPLIFICATION METHOD (HSAM) NANOSTRUCTURES FOR DIAGNOSTIC AND THERAPEUTIC USES	21/06/2002	in
19. MONO AND DUAL CONJUGATION OF NANOSTRUCTURES AND METHODS OF MAKING AND USING THEREOF	19/11/2002	in
20. CONTINUOUS PROCESS FOR THE ASSEMBLY OF MACROMOLECULAR SUBSTANCES AND THE SUBSEQUENT CAPTURE AND ISOLATION OF	26/08/2003	excl

MAMACROMOLECULAR ASSEMBLY AND A SYSTEM SUITABLE FOR THE PROCESS		
21. POLYGONAL NANOSTRUCTURES OF POLYNUCLEIC ACID MULTI-CROSSOVER MOLECULES AND ASSEMBLY OF LATTICES BASED ON DOUBLE CROSSOVER COHESION	10/06/2004	in
22. SELF-ASSEMBLED NUCLEIC ACID NANOARRAYS AND USES THEREFOR	12/05/2005	in
23. METHODS OF MAKING NUCLEIC ACID NANOSTRUCTURES	14/06/2005	in
24. NUCLEIC ACID NANOSTRUCTURE AND METHOD OF MANUFACTURING THE SAME	08/12/2005	in
25. POLYHEDRAL NANOSTRUCTURES FORMED FROM NUCLEIC ACIDS	20/04/2006	in
26. METHOD FOR SELF-ASSEMBLY OF ARBITRARY METAL PATTERNS ON DNA SCAFFOLDS	26/10/2006	in
27. WIREFRAME NANOSTRUCTURES	17/04/2007	in
28. NANOSTRUCTURES AND METHODS OF MAKING	14/05/2007	in
29. DNA MICROARRAY HAVING HAIRPIN PROBES TETHERED TO NANOSTRUCTURED METAL SURFACE	05/11/2007	in
30. METHODS AND KITS FOR NUCLEIC ACID SEQUENCING	03/09/2008	excl
31. METHOD FOR PRODUCING A NUCLEOPROTEIN NANOPARTICLE	01/10/2008	excl
32. NOVEL DNA NANOSTRUCTURES THAT PROMOTE CELL-CELL INTERACTION AND USE THEREOF	03/10/2008	in
33. SENSOR FOR DETECTION OF NUCLEIC ACID	20/11/2008	excl
34. TLR LIGAND-NUCLEIC ACID NANOSTRUCTURE AS A NOVEL IMMUNE MODULATORY AGENT AND METHOD OF USING THE SAME	24/11/2008	in
35. APPARATUS AND METHOD FOR MEASUREMENT USING NANOSTRUCTURE DEVICE	03/04/2009	excl
36. MULTI-SPOT METAL-CAPPED NANOSTRUCTURE ARRAY NUCLEIC ACID CH IP FOR DIAGNOSING OF CORNEAL DYSTROPHY AND PREPARATION METHOD THEREOF PRODUCING SAME	18/08/2009	in
37. MULTIFUNCTIONAL APTAMER-NUCLEIC ACID NANOSTRUCTURES FOR TUMOR-TARGETED KILLING	20/10/2009	in
38. NUCLEIC ACID NANOSTRUCTURE BARCODE PROBES	29/10/2010	in
39. METHOD AND APPARATUS FOR CONTROLLING PROPERTIES OF NUCLEIC ACID NANOSTRUCTURES	22/12/2010	in
40. SYSTEMS AND METHODS FOR HIGH RESOLUTION BIOMOLECULAR IMAGING AND ANALYSIS	11/03/2011	excl
41. FINITE FULLY ADDRESSABLE NUCLEIC ACID NANOSTRUCTURES AS	04/05/2011	in

NANOCARRIERS FOR DELIVERY OF PHARMACEUTICALS		
42. NANOPARTICLES HAVING PREDETERMINED SHAPES	01/07/2011	in
43. DEVELOPMENT OF NUCLEIC ACID GEL MATRIX FOR CELL-FREE PROTEIN SYNTHESIS OF CELL NUCLEUS REPLICATE, AND METHOD FOR PRODUCING SAME	17/11/2011	in
44. SELECTIVE NUCLEIC ACID AMPLIFICATION FROM NUCLEIC ACID POOLS	09/02/2012	in
45. USES OF IDED NANOSTRUCTURES IN NUCLEIC ACID TECHNOL OGY	19/02/2012	excl
46. LIPID-COATED NUCLEIC ACID NANOSTRUCTURES OF DEFINED SHAPE	26/03/2012	in
47. SELF-ASSEMBLY OF NUCLEIC ACID NANOSTRUCTURES	24/07/2012	in
48. FLUIDIC CHANNEL COATED WITH METAL CATALYSTS AND DEVICES AND METHODS RELATING THERETO	19/09/2012	excl
49. COMPOSITIONS AND METHODS RELATING TO COMPLEX NUCLEIC ACID NANOSTRUCTURES	06/11/2012	in
50. METHODS AND COMPOSITIONS FOR NANOSTRUCTURE-BASED NUCLEIC ACID SEQUENCING	20/02/2013	excl
51. COMPOSITIONS AND METHODS RELATING TO NUCLEIC ACID-PROTEIN COMPLEXES	10/07/2013	in
52. FABRICATION OF HIERARCHICAL SILICA NANOMEMBRANES AND USES THEREOF FOR SOLID PHASE EXTRACTION OF NUCLEIC ACIDS	05/08/2013	excl
53. METHOD FOR DETECTING ENDONUCLEASE DNASEI ACTIVITY BASED ON CHIRAL GOLD TETRAHEDRAL NANOSTRUCTURE	21/08/2013	in
54. ENGINEERING DNA ASSEMBLY IN VIVO AND METHODS OF MAKING AND USING THE REVERSE TRANSCRIPTASE TECHNOLOGY	26/09/2013	in
55. NUCLEIC ACID NANOSTRUCTURES FOR IN VIVO AGENT DELIVERY	08/11/2013	in
56. CGAP-PNA MULTIVALENT PEPTIDE NUCLEIC ACID LIGAND DISPLAY	21/01/2014	excl
57. NONDESTRUCTIVE COLLECTION OF EVIDENCE	10/02/2014	excl
58. NANOSTRUCTURE AND METHODS OF NUCLEIC ACID ISOLATION	09/03/2014	excl
59. METHOD OF PREPARING BASE SEQUENCE OF 5'-COHESIVE END FOR SYNTHESIZING BRANCHED NUCLEIC ACID NANOSTRUCTURE	01/04/2014	in
60. SELF ASSEMBLING NUCLEIC ACID NANOSTRUCTURES	03/04/2014	in

61. SELF-ASSEMBLY OF DNA ORIGAMI: A NEW DIAGNOSTIC TOOL	29/04/2014	in
62. TRIANGULAR NUCLEIC ACID NANO-PREPARATION, AND PREPARATION METHOD AND APPLICATION THEREOF	07/05/2014	in
63. LEFT-HANDED GAMMA-PEPTIDE NUCLEIC ACIDS, METHODS OF SYNTHESIS AND USES THEREFOR	08/05/2014	excl
64. SCALABLE NUCLEIC ACID-BASED NANOFABRICATION	22/05/2014	in
65. NUCLEIC ACID AMPHIPHILES AND NANOSTRUCTURES	23/06/2014	in
66. DRUG CARRIER HAVING SELF-ASSEMBLED 3-D NUCLEIC ACID NANOSTRUCTURE	23/07/2014	in
67. IMPROVED NANOPORE PLASMONIC ANALYSER	15/09/2014	excl
68. TECHNIQUES FOR CONTROLLING SPATIAL STRUCTURE OF NUCLEIC ACID STRUCTURES BASED ON LATTICE-FREE, THREE DIMENSIONAL JUNCTION COORDINATES	03/10/2014	in
69. METHOD FOR MASS PRODUCTION OF NUCLEIC ACID NANOSTRUCTURE AND USE THEREOF IN DRUG DELIVERY SYSTEMS	21/10/2014	in
70. METAFLUOROPHORES	16/12/2014	in
71. NUCLEIC ACID NANOSTRUCTURES WITH CORE MOTIFS	14/01/2015	in
72. SINGLE-STRANDED DNA NANOSTRUCTURES	07/03/2015	in
73. METHODS OF FORMING NANOSTRUCTURES USING SELF-ASSEMBLED NUCLEIC ACIDS, AND NANOSTRUCTURES THEREOF	02/04/2015	excl
74. BRANCHED NUCLEIC ACID NANOSTRUCTURE HA VING IMPROVED EFFICIENCY OF FLUORESCENCE ANALYSIS FOR FLOW CYTOMETRY AND MANUFACTURING METHOD THEREOF	08/06/2015	in
75. STRUCTURAL NUCLEIC ACID NANOTECHNOLOG Y IN ENERGY APPLICATIONS	28/08/2015	in
76. NUCLEIC ACID FRAMEWORKS FOR STRUCTURAL DETERMINATION	18/09/2015	in
77. METHOD FOR SELF-ASSEMBLING MULTI-CHAIN NUCLEIC ACID MODULES INTO LIMITED NUCLEIC ACID NANOSTRUCTURE	25/09/2015	in
78. NANOSTRUCTURES WITH CATALYTIC ACTIVITY	27/11/2015	in
79. ANTI-MICROBIAL COMPOSITIONS COMPRISING NUCLEIC ACID NANOSTRUCTURES AND METHODS OF MAKING AND USING SUCH COMPOSITIONS	08/01/2016	in
80. SEQUENCE DESIGN FOR EFFICIENT ASSEMBLY OF NUCLEIC ACID STRUCTURES	11/04/2016	in
81. SELF-ASSEMBLED, ELECTRONICALLY-FUNCTIONAL NUCLEIC ACID NANOSTRUCTURES AND	11/04/2016	in

NETWORKS BASED ON THE USE OF ORTHOGONAL BASE PAIRS		
82. NOVEL NUCLEIC ACID NETWORK NANOSTRUCTU RE BASED ON TOPOISOMERASE AND PREPARATION METHOD THEREOF	19/04/2016	in
83. STABLE NANOSCALE NUCLEIC ACID ASSEMBLIES AND METHODS THEREOF	27/04/2016	in
84. SEQUENCE-CONTROLLED POLYMER RANDOM ACCESS MEMORY STORAGE	27/04/2016	in
85. FUNCTIONALIZED NUCLEIC ACID NANOSTRUCTU RES FOR RNA DELIVERY	20/07/2016	in
86. DUAL-MODE SELF-ASSEMBLY SUPRAMOLECULAR NUCLEOPROTEIN NANOSTRUCTURE AND THE METHOD OF PREPARING THE COMPOUND	05/08/2016	in
87. HIGH THROUGHPUT OIL-EMULSION SYNTHESIS OF BOWTIE BARCODES FOR PAIRED MRNA CAPTURE AND SEQUENCING FROM INDIVIDUAL CELLS	19/08/2016	in
88. METHOD FOR PREPARING FINITE OR INFINITE DNA NANOSTRUCTURES THROUGH SELF-ASSEMBLING OF NUCLEIC ACID STRUCTURAL UNITS	31/08/2016	in
89. NUCLEIC ACID DETECTION METHOD AND KIT	23/09/2016	in
90. METHOD OF PREPARING AMPLIFIED APTAMER NANOCONSTURCTS BASED ON DEXTRAN POLYMER FOR SELECTIVELY CAPTURING TARGET PROTEIN	01/11/2016	in
91. TDNS-AS1411-NUCLEIC ACID DRUG NANOCOMPOSITE BASED DRUG DELIVERY SYSTEM AND PREPARATION METHOD THEREOF	02/11/2016	in
92. INTEGRATED ELECTROCHEMICAL NUCLEIC ACID BASED SENSORS AND RELATED PLATFORMS	13/02/2017	in
93. OPTICAL AND OPTO-ACOUSTIC INTEGRATION DUAL-MODE MOLECULAR IMAGE PROBE AS WELL AS PREPARATION METHOD AND APPLICATION THEREOF	16/02/2017	in
94. STIMULI-RESPONSIVE TYPE NUCLEIC ACID NANOSTRUCTURE CARRIER CHIRAL NOBLE METAL NANOCOMPOSITE AND PREPARATION METHOD THEREOF AND APPLICATION	20/02/2017	in
95. NANOSTRUCTURE WITH A NUCLEIC ACID SCAFFOLD AND VIRUS-BINDING PEPTIDE MOIETIES	26/05/2017	in
96. METHOD FOR STEPWISE ASSEMBLING DNA ORIGAMI UNITS	02/06/2017	in
97. PORTABLE SINGLE-MOLECULE BIO-SENSING DEVICE	16/06/2017	excl
98. COMPOSITIONS AND METHODS FOR DETECTING VIRAL NUCLEIC ACIDS	23/06/2017	in

99. BRANCHED NUCLEIC ACID NANOSTRUCTURE HA VING IMPROVED EFFICIENCY OF FLUORESCENCE ANALYSIS FOR FLOW CYTOMETRY AND MANUFACTURING METHOD THEREOF	24/07/2017	dup
100. NUCLEIC ACID AND OTHER COMPOSITIONS AND METHODS FOR THE MODULATION OF CELL MEMBRANES	02/11/2017	in
101. PHOTOELECTROCHEMICAL NUCLEIC ACID ANA LYSIS METHOD BASED ON DEFECT-REGULATED SEMICONDUCTOR	05/12/2017	in
102. COMPOSITIONS FOR THE TREATMENT OR PREVENTION OF NEURODEGENERATIVE DISORDERS, IN PARTICULAR PARKINSON`S DISEASE	11/12/2017	excl
103. SIGNAL AMPLIFICATION USING NUCLEIC ACID NANOSTRUCTURE LINKED BY GOLD NANOPARTICLE AND ITS APPLICATIONS	29/12/2017	in
104. - GRAPHENE BIOSENSOR WITH FLUORESCENCE-LABELED NUCLEIC ACID NANOSTRUCTURE FOR DETECTING NUCLEIC ACID	12/01/2018	n/a
105. A SET OF ANTI-PATHOGENIC NUCLEIC ACIDS, COMPOSITIONS AND USES THEREOF	17/01/2018	n/a
106. SELF-ASSSEMBLING NANOSTRUCTURE VACCINES	28/02/2018	n/a
107. ASPNA (ANTISENSE PEPTIDE NUCLEIC ACID)-TDN (TETRAHEDRAL DNA NANOSTRUCTURE) CARRIER COMPOUND AS WELL AS PREPARATION METHOD AND APPLICATION THEREOF	13/04/2018	n/a
108. NUCLEIC ACID PROTEIN NANOCOMPOSITE AS WELL AS PREPARATION METHOD AND APPLICATION THEREOF	23/04/2018	n/a
109. ELECTROCHEMICAL SENSOR FOR DETECTING MICRORNA-21, PREPARATION METHOD AND APPLICATION THEREOF	13/12/2018	n/a
110. BRANCHED NUCLEIC ACID NANOSTRUCTURE H AVING IMPROVED EFFICIENCY OF FLUORESCENCE ANALYSIS FOR FLOW CYTOMETRY AND MANUFACTURING METHOD THEREOF	18/12/2018	n/a
111. - FUSION NANO LIPOSOME-NUCLEIC ACID FOR QUANTITATIVE DIAGNOSIS OF MULTI RIBONUCLEIC ACID MARKER METHOD FOR THEORETICAL STABILITY EVALUATION THEREOF USES THEREOF AND PREPARATION METHOD THEREOF	15/02/2019	n/a

Supplementary Data 2: Further analysis of DNA origami patent applications

Numbering of patents matches table in Supplementary Data 1.

*No. of inventors: this is the number of people named as inventors on the patent application. Note that this is not necessarily the same as the number of people listed as co-authors on the corresponding scientific paper. The threshold for inclusion as an inventor on a patent application is usually higher than the threshold for inclusion in the author list of a paper.

**Nationality: this is the nationality of the inventor, where given. Where the nationality is not given in the patent application, the nationality of the originating institution is used.

US – United States; CN – China; KR – South Korea; DE – Germany; JP – Japan; TR – Turkey; FR – France; IL – Israel

***Source: academia (A), industry (I) or both (A+I). This was primarily determined based on the name of the applicant. For instance 'X Univ' or 'Institute of Y' would be academic institutions. A name ending in 'Co Ltd' was classified as I.

**Title**	**Priority date**	**No. of inventors***	**Nationality****	**Source*****
1. NUCLEIC ACID NANOTUBE LIQUID CRYSTALS AND USE FOR NMR STRUCTURE DETERMINATION OF MEMBRANE PROTEINS	21/04/2006	3	US	A
2. CONTROLLABLE DISTRIBUTION METHOD OF GOLD NANOPARTICLES ON DNA ORIGAMI CHIP	12/10/2010	4	CN	A+I
6. DNA ORIGAMI DEVICES	04/11/2010	3	1 IL + 2 US	A
7. METHOD AND APPARATUS FOR CONTROLLING PROPERTIES OF NUCLEIC ACID NANOSTRUCTURES	22/12/2010	3	2US + 1DE	A
8. REAL TIME IN SITU CHARACTERIZATION METHOD FOR SINGLE BIOMOLECULAR REACTION	22/03/2011	3	CN	A
10. NOVEL DNA-ORIGAMI NANOVACCINES	06/02/2012	3	US	A
11. SPATIAL SEQUENCING OF NUCLEIC ACIDS USING DNA ORIGAMI PROBES	05/06/2012	3	US	A
12. DNA-ORIGAMI-BASED STANDARD	22/08/2012	3	DE	A
14. BIOMOLECULE AFFINITY CONSTANT DETERMINATION METHOD BASED ON DNA (DEOXYRIBONUCLEIC ACID) ORIGAMI	15/10/2012	4	CN	A
15. A METHOD TO PCR AMPLIFY MULTIPLE SEQUENCE ELEMENTS THAT ARE DISTANTLY LOCATED ON A SINGLE	13/12/2012	2	KR	A

NUCLEIC ACID TOGETHER INTO A SINGLE AMPLICON				
16. METHODS FOR OBTAINING INFORMATION FROM SINGLE CELLS WITHIN POPULATIONS USING DNA ORIGAMI NANOSTRUCTURES WITHOUT THE NEED FOR SINGLE CELL SORTING	12/06/2013	4	US	A
17. COMPOSITIONS AND METHODS RELATING TO NUCLEIC ACID-PROTEIN COMPLEXES	10/07/2013	2	US	A
18. NUCLEIC ACID NANO STRUCTURE FOR CARRYING ANTITUMOR DRUGS, PREPARATION METHOD AND APPLICATIONS THEREOF	13/08/2013	5	CN	A
19. SELF-ASSEMBLY OF DNA ORIGAMI: A NEW DIAGNOSTIC TOOL	29/04/2014	1	DE	I
20. DETECTION METHOD USING DNA NANO-ORIGAMI STRUCTURE AS SIGNAL AMPLIFICATION PROBE	28/05/2014	4	CN	I
21.MECHANOCHEMICAL PLATFORM AND SENSING METHODS USING DNA ORIGAMI NANOSTRUCTURES	06/06/2014	4	2 US 2JP	A
22. SITE-SPECIFIC IMMOBILIZATION OF DNA ORIGAMI STRUCTURES ON SOLID SUBSTRATES	14/10/2014	8	7DE + 1 FR	A
24. METHOD FOR SIMULATION OF DNA NANO ORIGAMI STRUCTURE AS DRUG CARRIER BY DAPI EMBEDDING AND RELEASE	26/06/2015	4	CN	I
25. A PREPARATION METHOD FOR A DNA ORIGAMI BASED CARRIER SYSTEM	22/01/2016	1	TR	A
26. METHOD FOR DETECTING INFLUENCE OF MECHANICAL FORCE ON INTERACTION OF DNA (DEOXYRIBOSE NUCLEIC ACID) AND DNA POLYMERASE	05/02/2016	5	CN	A
27. SAMPLE PREPARATION METHOD EMPLOYING AFM (ATOMIC FORCE MICROSCOPE) FOR SINGLE ANTIBODY MOLECULE IMAGING	07/04/2016	8	CN	A
28. METHOD FOR CONSTRUCTING DOLMEN STRUCTURE BASED ON DNA ORIGAMI TEMPLATE AND GOLD NANORODS	22/12/2016	4	CN	A
29. SYSTEMS AND METHODS FOR DETERMINING MOLECULAR MOTION	05/01/2017	5	US	A
30. OPTICAL AND OPTO-ACOUSTIC INTEGRATION DUAL-MODE MOLECULAR	16/02/2017	6	CN	A

IMAGE PROBE AS WELL AS PREPARATION METHOD AND APPLICATION THEREOF				
32. DNA ORIGAMI NANOSTRUCTURES FOR TREATMENT OF ACUTE KIDNEY INJURY	09/03/2017	8	5US, 1KR, 2CN	A
33. DNA MOLECULAR LOGIC GATE BASED ON DNA ORIGAMI AND CONSTRUCTION METHOD THEREOF	23/03/2017	4	CN	A
34. NOVEL MICRO-ENVIRONMENT BIOLOGICAL MACROMOLECULE GENERAL OSCILLATOR AS WELL AS SYNTHESIS METHOD AND APPLICATION THEREOF	24/04/2017	6	CN	A
35. METHOD FOR STEPWISE ASSEMBLING DNA ORIGAMI UNITS	02/06/2017	4	CN	A
36. DNA ORIGAMI-BASED CONSTRUCTION METHOD AND APPLICATION OF PRECISE RECOGNITION TARGETED NANO-CARRIER	27/06/2017	4	CN	A
37. PROBE MACHINE ACHIEVEMENT METHOD AND DEVICE BASED ON DNA COMPUTING	17/07/2017	4	CN	A
38. NANOPROBE USED FOR SPECIFIC RECOGNITION OF DNA SEQUENCES, AND APPLICATION METHOD THEREOF	19/07/2017	4	CN	A
39. METHOD FOR DETERMINING SIGNAL ACQUISITION SITE UNDER SINGLE MOLECULE FLUORESCENCE OBSERVATION BASED ON DNA ORIGAMI	10/08/2017	7	CN	I
40. METHOD FOR STUDYING CASCADE REACTIONS UNDER SINGLE-MOLECULE CONDITION	10/08/2017	9	CN	I
41. FLUORESCENCE PRECISE QUANTIFICATION METHOD OF INTEGRITY OF DNA ORIGAMI STRUCTURE	27/10/2017	6	CN	I
43. METHOD OF DETECTING TARGET SARCOSINE OF PROSTATIC CANCER	07/11/2017	5	CN	I
44. MICROBIAL PRODUCTION OF PURE SINGLE STRANDED NUCLEIC ACIDS	10/11/2017	3	US	A
45. DNA NANO-IMAGING PROBE FOR SUBCELLULAR-SCALE CELLULAR CO-LOCALIZATION AS WELL AS PREPARATION METHOD AND APPLICATION OF DNA NANO-IMAGING PROBE	28/11/2017	6	CN	I
46. DNA NANOROBOT AND METHODS OF USE THEREOF	07/12/2017	5	1 US and 4CN	A

48. DNA ORIGAMI NANOPARTICLE DELIVERY OF PROGRAMMED CHROMOSOME BREAKAGE MACHINERY	22/12/2017	2	US	A
49. MANUFACTURING METHOD OF DNA-BASED CONDUCTIVE COATING AND PRODUCT AND USE THEREOF	26/12/2017	6	CN	I
51. MOLECULAR LOGIC GATE BASED ON DNA ORIGAMI AND NANO METAL PARTICLE FLUORESCENT PROBE	27/12/2017	3	CN	A

<u>Supplementary Data 3:</u> Using Web of Science to analyse the affiliations of authors of DNA nanotechnology papers

A search was performed with Web of Science on 7th January 2020, for papers on the topic of "DNA nanotechnology", with " and " included in the search box. This table includes only those organizations associated with three or more records.

	Organization	Number of records
1	CHINESE ACADEMY OF SCIENCES	59
2	NEW YORK UNIVERSITY	50
3	ARIZONA STATE UNIVERSITY	47
4	HARVARD UNIVERSITY	45
5	UNIVERSITY OF CALIFORNIA SYSTEM	43
6	TECHNICAL UNIVERSITY OF MUNICH	36
7	SHANGHAI INSTITUTE OF APPLIED PHYSICS CAS	27
8	UNIVERSITY OF MUNICH	26
9	AARHUS UNIVERSITY	24
10	CALIFORNIA INSTITUTE OF TECHNOLOGY	24
11	UNIVERSITY OF OXFORD	21
12	PURDUE UNIVERSITY	20
13	PURDUE UNIVERSITY SYSTEM	20
14	UNIVERSITY OF NORTH CAROLINA	18
15	DANA FARBER CANCER INSTITUTE	17
16	KYOTO UNIVERSITY	17
17	NORTH CAROLINA STATE UNIVERSITY	17
18	UNIVERSITY OF CAMBRIDGE	17
19	DUKE UNIVERSITY	16
20	BOISE STATE UNIVERSITY	15
21	MASSACHUSETTS INSTITUTE OF TECHNOLOGY MIT	15
22	UNIVERSITY OF ROME TOR VERGATA	15
23	MCGILL UNIVERSITY	14
24	UNIVERSITY OF MICHIGAN	14
25	UNIVERSITY OF MICHIGAN SYSTEM	14
26	OHIO STATE UNIVERSITY	13
27	SUNGKYUNKWAN UNIVERSITY SKKU	13
28	UNIVERSITY OF CALIFORNIA SANTA BARBARA	13
29	UNIVERSITY OF ILLINOIS SYSTEM	13
30	HEBREW UNIVERSITY OF JERUSALEM	12
31	HUAZHONG UNIVERSITY OF SCIENCE TECHNOLOGY	12
32	MAX PLANCK SOCIETY	12
33	UNIVERSITY OF ILLINOIS URBANA CHAMPAIGN	11
34	AALTO UNIVERSITY	10
35	DRESDEN UNIVERSITY OF TECHNOLOGY	10
36	UNIVERSITY OF CHINESE ACADEMY OF SCIENCES CAS	10
37	UNIVERSITY OF TOKYO	10
38	NATIONAL CENTER FOR NANOSCIENCE TECHNOLOGY CHINA	9

39	PEKING UNIVERSITY	9
40	STATE UNIVERSITY SYSTEM OF FLORIDA	9
41	UNITED STATES DEPARTMENT OF ENERGY DOE	9
42	UNIVERSITY OF MONTREAL	9
43	UNIVERSITY OF SCIENCE TECHNOLOGY OF CHINA	9
44	EMORY UNIVERSITY	8
45	GEORGIA INSTITUTE OF TECHNOLOGY	8
46	HUNAN UNIVERSITY	8
47	KAROLINSKA INSTITUTET	8
48	STATE UNIVERSITY OF NEW YORK SUNY SYSTEM	8
49	TSINGHUA UNIVERSITY	8
50	UNIVERSITY OF LONDON	8
51	UNIVERSITY OF SOUTHAMPTON	8
52	UNIVERSITY OF TEXAS SYSTEM	8
53	UNIVERSITY SYSTEM OF GEORGIA	8
54	CENTRE NATIONAL DE LA RECHERCHE SCIENTIFIQUE CNRS	7
55	CHALMERS UNIVERSITY OF TECHNOLOGY	7
56	HARVARD MEDICAL SCHOOL	7
57	HELMHOLTZ ASSOCIATION	7
58	IMPERIAL COLLEGE LONDON	7
59	JAPAN SCIENCE TECHNOLOGY AGENCY JST	7
60	NANJING UNIVERSITY OF POSTS TELECOMMUNICATIONS	7
61	NATIONAL CENTRE FOR BIOLOGICAL SCIENCES NCBS	7
62	PENNSYLVANIA COMMONWEALTH SYSTEM OF HIGHER EDUCATION PCSHE	7
63	SHANGHAI JIAO TONG UNIVERSITY	7
64	TATA INSTITUTE OF FUNDAMENTAL RESEARCH TIFR	7
65	UNITED STATES DEPARTMENT OF DEFENSE	7
66	UNIVERSITY OF BONN	7
67	UNIVERSITY OF WASHINGTON	7
68	UNIVERSITY OF WASHINGTON SEATTLE	7
69	COLUMBIA UNIVERSITY	6
70	HONG KONG UNIVERSITY OF SCIENCE TECHNOLOGY	6
71	NANOSYST INITIAT MUNICH	6
72	NATIONAL UNIVERSITY OF SINGAPORE	6
73	SAPIENZA UNIVERSITY ROME	6
74	UNITED STATES NAVY	6
75	UNIVERSITY COLLEGE LONDON	6
76	UNIVERSITY OF CALIFORNIA RIVERSIDE	6
77	UNIVERSITY OF MARYLAND COLLEGE PARK	6
78	UNIVERSITY OF TEXAS AUSTIN	6
79	UNIVERSITY SYSTEM OF MARYLAND	6
80	BIRKBECK UNIVERSITY LONDON	5
81	BRAUNSCHWEIG UNIVERSITY OF TECHNOLOGY	5
82	CHANGCHUN INSTITUTE OF APPLIED CHEMISTRY CAS	5

83	CHINA UNIVERSITY OF PETROLEUM	5
84	CORNELL UNIVERSITY	5
85	JAPAN ADVANCED INSTITUTE OF SCIENCE TECHNOLOGY JAIST	5
86	KARLSRUHE INSTITUTE OF TECHNOLOGY	5
87	NAGOYA UNIVERSITY	5
88	NANJING UNIVERSITY	5
89	NAVAL RESEARCH LABORATORY	5
90	STATE UNIVERSITY OF NEW YORK SUNY ALBANY	5
91	TOKYO INSTITUTE OF TECHNOLOGY	5
92	UNIVERSITY OF CALIFORNIA BERKELEY	5
93	UNIVERSITY OF CALIFORNIA LOS ANGELES	5
94	UNIVERSITY OF CALIFORNIA SAN DIEGO	5
95	UNIVERSITY OF CENTRAL FLORIDA	5
96	UNIVERSITY OF CHICAGO	5
97	UNIVERSITY OF CINCINNATI	5
98	UNIVERSITY OF GRONINGEN	5
99	UNIVERSITY OF PITTSBURGH	5
100	WUHAN UNIVERSITY	5
101	BAR ILAN UNIVERSITY	4
102	BEN GURION UNIVERSITY	4
103	CNRS INSTITUTE OF CHEMISTRY INC	4
104	DORTMUND UNIVERSITY OF TECHNOLOGY	4
105	GEORGE MASON UNIVERSITY	4
106	GOETHE UNIVERSITY FRANKFURT	4
107	INSTITUT NATIONAL DE LA SANTE ET DE LA RECHERCHE MEDICALE INSERM	4
108	INSTITUTE OF CHEMISTRY CAS	4
109	JOHNS HOPKINS UNIVERSITY	4
110	KANSAI UNIVERSITY	4
111	NATIONAL INSTITUTES OF HEALTH NIH USA	4
112	NORTHWESTERN UNIVERSITY	4
113	OSAKA UNIVERSITY	4
114	QINGDAO UNIVERSITY OF SCIENCE TECHNOLOGY	4
115	RICE UNIVERSITY	4
116	SOUTHWEST UNIVERSITY CHINA	4
117	TOHOKU UNIVERSITY	4
118	UCB PHARMA SA	4
119	UNIVERSITY OF ALBERTA	4
120	UNIVERSITY OF CALIFORNIA DAVIS	4
121	UNIVERSITY OF MILAN	4
122	UNIVERSITY OF TRIESTE	4
123	ARMY MEDICAL UNIVERSITY	3
124	BROOKHAVEN NATIONAL LABORATORY	3
125	CENTRAL SOUTH UNIVERSITY	3
126	CONSEJO SUPERIOR DE INVESTIGACIONES CIENTIFICAS CSIC	3

127	COUNCIL OF SCIENTIFIC INDUSTRIAL RESEARCH CSIR INDIA	3
128	CSIR ACADEMY OF SCIENTIFIC INNOVATIVE RESEARCH ACSIR	3
129	EAST CHINA NORMAL UNIVERSITY	3
130	FUDAN UNIVERSITY	3
131	HOWARD HUGHES MEDICAL INSTITUTE	3
132	IOWA STATE UNIVERSITY	3
133	KOREA ADVANCED INSTITUTE OF SCIENCE TECHNOLOGY KAIST	3
134	KOREA INSTITUTE OF SCIENCE TECHNOLOGY KIST	3
135	LAWRENCE BERKELEY NATIONAL LABORATORY	3
136	MARSHALL UNIVERSITY	3
137	MISSOURI UNIVERSITY OF SCIENCE TECHNOLOGY	3
138	NATIONAL INSTITUTE OF CHEMISTRY SLOVENIA	3
139	NIH NATIONAL CANCER INSTITUTE NCI	3
140	NORTHEASTERN UNIVERSITY	3
141	OCEAN UNIVERSITY OF CHINA	3
142	QINGDAO UNIVERSITY	3
143	RUPRECHT KARLS UNIVERSITY HEIDELBERG	3
144	SICHUAN UNIVERSITY	3
145	SORBONNE UNIVERSITE	3
146	STANFORD UNIVERSITY	3
147	TEL AVIV UNIVERSITY	3
148	UNIVERSITA CA FOSCARI VENEZIA	3
149	UNIVERSITE DE MONTPELLIER	3
150	UNIVERSITE PARIS SACLAY	3
151	UNIVERSITY OF CALIFORNIA IRVINE	3
152	UNIVERSITY OF FLORIDA	3
153	UNIVERSITY OF MINNESOTA SYSTEM	3
154	UNIVERSITY OF MINNESOTA TWIN CITIES	3
155	UNIVERSITY OF MISSOURI SYSTEM	3
156	UNIVERSITY OF NEBRASKA SYSTEM	3
157	UNIVERSITY OF NEW SOUTH WALES SYDNEY	3
158	UNIVERSITY OF YORK UK	3
159	YALE UNIVERSITY	3
160	YONSEI UNIVERSITY	3
161	ZHENGZHOU UNIVERSITY OF LIGHT INDUSTRY	3
